# The Ebola virus VP35 protein binds viral immunostimulatory and host RNAs identified through deep sequencing

**DOI:** 10.1371/journal.pone.0178717

**Published:** 2017-06-21

**Authors:** Kari A. Dilley, Alexander A. Voorhies, Priya Luthra, Vinita Puri, Timothy B. Stockwell, Hernan Lorenzi, Christopher F. Basler, Reed S. Shabman

**Affiliations:** 1 Virology Group, J. Craig Venter Institute, Rockville, Maryland, United States of America; 2 Infectious Disease Group, J. Craig Venter Institute, Rockville, Maryland, United States of America; 3 Center for Microbial Pathogenesis, Georgia State University, Atlanta, Georgia, United States of America; Louisiana State University, UNITED STATES

## Abstract

Ebola virus and Marburg virus are members of the *Filovirdae* family and causative agents of hemorrhagic fever with high fatality rates in humans. Filovirus virulence is partially attributed to the VP35 protein, a well-characterized inhibitor of the RIG-I-like receptor pathway that triggers the antiviral interferon (IFN) response. Prior work demonstrates the ability of VP35 to block potent RIG-I activators, such as Sendai virus (SeV), and this IFN-antagonist activity is directly correlated with its ability to bind RNA. Several structural studies demonstrate that VP35 binds short synthetic dsRNAs; yet, there are no data that identify viral immunostimulatory RNAs (isRNA) or host RNAs bound to VP35 in cells. Utilizing a SeV infection model, we demonstrate that both viral isRNA and host RNAs are bound to Ebola and Marburg VP35s in cells. By deep sequencing the purified VP35-bound RNA, we identified the SeV copy-back defective interfering (DI) RNA, previously identified as a robust RIG-I activator, as the isRNA bound by multiple filovirus VP35 proteins, including the VP35 protein from the West African outbreak strain (Makona EBOV). Moreover, RNAs isolated from a VP35 RNA-binding mutant were not immunostimulatory and did not include the SeV DI RNA. Strikingly, an analysis of host RNAs bound by wild-type, but not mutant, VP35 revealed that select host RNAs are preferentially bound by VP35 in cell culture. Taken together, these data support a model in which VP35 sequesters isRNA in virus-infected cells to avert RIG-I like receptor (RLR) activation.

## Importance

Ebola virus and Marburg virus infection is characterized by widespread immune dysregulation resulting in high mortality rates. Disease severity often correlates with an ability of the virus to suppress innate immune responses following infection. VP35 is a robust inhibitor of the host innate immune responses, derailing the cell’s first line of antiviral defense. The ability of VP35 to inhibit host immunity is tightly linked to its ability to bind RNA, though what RNA species are bound in virus-infected cells has been undefined. Here, we demonstrate for the first time that *Ebolavirus* and *Marburgvirus* VP35s bind viral immunostimulatory RNA in infected cells. Moreover, we serendipitously discovered that VP35 also binds select host RNAs in cells, suggesting its ability to interact with both viral and host cell RNA upon infection. Our data support a model in which VP35 sequesters viral RNA in infected cells to preempt activation of antiviral responses.

## Introduction

The family *Filoviridae*, which includes Ebola virus (EBOV) and Marburg virus (MARV), are filamentous viruses with a single-stranded, non-segmented, negative-sense RNA genome [1]. Filovirus infection of humans and non-human primates can result in severe hemorrhagic fever with high mortality rates [1]. EBOV and MARV infections are generally limited to isolated outbreaks. However, since 2013, an EBOV outbreak in West Africa has resulted in wide-spread human-to-human EBOV transmission [2] with over 28,600 suspected, probable and confirmed cases and over 11,300 deaths, highlighting the importance of understanding factors that influence filoviral virulence and pathology (reviewed in [3,4]).

Filovirus pathogenesis is characterized by unrestrained virus replication, a “cytokine storm”, coagulopathy, and lack of an adaptive immune response (Reviewed in [5–7]). Furthermore, filovirus virulence correlates with an ability to suppress host innate immune responses, a first line of defense against viral infection, and a prerequisite for induction of the adaptive immune response. Type I interferons (IFN-α/β), central components of the innate immune response, are activated by viral RNA through members of the RIG-I-like receptor (RLR) family of cytoplasmic RNA sensors, including RIG-I and MDA5 [8] (reviewed in 9). In general, RIG-I recognizes viral uncapped double-stranded RNA (dsRNA) with a 5’ triphosphate, while MDA5 is believed to be activated by longer dsRNA structures (Reviewed in [9]). Importantly, EBOV genomic RNA, in the absence of any viral proteins, is sufficient to trigger RIG-I activation, and upregulation of RIG-I in EBOV-infected cells significantly impairs the virus, reducing replication approximately 1000-fold [10,11].

Filoviruses encode multiple type I IFN antagonists. The EBOV and MARV VP35 proteins inhibit type I IFN (IFN-α/β) production, while the EBOV VP24 and MARV VP40 proteins block type I IFN signaling pathways [12–14]. Several studies demonstrate that EBOV and MARV VP35 suppress type I IFN production by impairing IRF-3 phosphorylation both through their ability to bind dsRNA and through direct interactions with the host proteins TBK-1, IKKε, and PACT [13,15–17]. The importance of VP35 dsRNA-binding activity is further emphasized by EBOV mouse and guinea pig models that are rendered avirulent by point mutations in the VP35 C-terminal dsRNA-binding domain [18,19].

Previous work indicates VP35 binds synthetic dsRNA molecules *in vitro* and can inhibit IFN induction mediated by these dsRNAs in cell culture [16,19,20]. However, direct evidence that VP35 binds immunostimulatory RNA (isRNA) of viral origins to impair RIG-I activation has not been demonstrated. In this study we aimed to survey viral isRNA and host RNAs associated with VP35 in cell culture. To this end, we purified EBOV and MARV VP35 in transfected 293T cells in the presence or absence of Sendai virus (SeV). SeV infection generates an excess of “copy-back” sub-genomic RNAs, termed defective interfering (DI) RNAs, which are potent inducers of RIG-I signaling and have been historically used to induce and study the IFN pathway [21,22]. We found that RNAs isolated from immunoprecipitated VP35 from SeV-infected cells were potent inducers of a type I IFN response. Deep sequencing of the VP35-bound RNAs demonstrated that VP35 binds the SeV DI sub-genomic RNAs shown to be activators of RIG-I antiviral signaling [9,23]. Furthermore, the ability of VP35 to bind the SeV DI RNA was ablated by mutating basic residues (K309 and R312) required for dsRNA binding and IFN inhibition. A panel of additional VP35 proteins, including the VP35 from the 2014 EBOV outbreak Makona strain, indicated that both *Ebolavirus* and *Marburgvirus* VP35s are able to bind the SeV DI RNA and inhibit induction of the IFN response. Finally, we surveyed host RNAs bound to a wild-type and a mutant EBOV VP35 through deep sequencing. We identify select host RNAs reproducibly bound by VP35 and hypothesize these may produce secondary structures similar to the SeV DI RNA. Taken together, this is the first study to identify viral and host RNAs directly bound to VP35 in cells, work that supports a model for EBOV RIG-I evasion and suggests that EBOV genomic isRNA may be bound by VP35 in EBOV-infected cells.

## Results

### The VP35 proteins from *Marburgvirus* and all *Ebolavirus* species antagonize SeV-induced IFN-β gene expression

There is strong evidence that the RNA-binding domain of VP35 from *Ebolavirus* and *Marburgvirus* is essential for inhibiting host type I IFN responses [17,19,20,24,25]. This activity has been historically modeled using a well-established IFN reporter assay where a reporter gene (luciferase, CAT, or GFP) placed under the control of the IFN-β promoter is co-transfected with a putative viral IFN antagonist or appropriate control plasmids. Transfected cells are either mock-infected or infected with SeV. In the absence of an IFN antagonist (such as a viral factor), SeV infection stimulates IFN-β promoter activity, while in the presence of an IFN antagonist, SeV-mediated IFN-β promoter activity will be inhibited. While this robust assay has evaluated select VP35 proteins, there is no data directly comparing all VP35 species’ ability to antagonize IFN. Therefore, this assay provides an ideal platform to directly compare VP35 IFN antagonist activity across filoviruses and mechanistically define viral isRNA bound by VP35.

**Fig 1:**
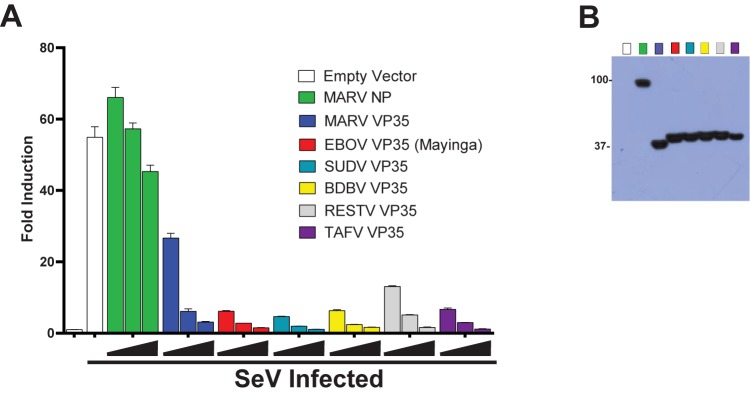
VP35 proteins from Marburg virus and all five *Ebolavirus* species antagonize SeV induced promoter activity. The abilities of the VP35 proteins from Marburgvirus and the five species of Ebolavirus to antagonize the IFN response induced by virus infection were compared in the context of a luciferase reporter under the control of the IFN-β promoter. (A) 293T cells were transfected with increasing amounts (4 ng, 20 ng, or 100 ng) of pCAGGS-based plasmids expressing N-terminally FLAG-tagged filoviral proteins, an empty pCAGGS plasmid to transfect equal amounts between samples, and a plasmid expressing Renilla luciferase as a transfection control. The following day, cells were either mock-infected or infected with SeV to induce IFN-β promoter activity. The third day, cells were harvested and luciferase expression was measured. Error bars represent standard error of the mean of triplicates. MARV, Marburg Virus; EBOV, Ebola Virus (Mayinga); SUDV, Sudan Virus; BDBV, Bundibugyo Virus; RESTV, Reston Virus; TAFV, Taï Forest Virus. (B) Western blot analysis against the FLAG tag shows relative expression of filoviral proteins when 100 ng of each FLAG-tagged protein-expressing plasmid was transfected.

We evaluated the ability of FLAG-tagged VP35 from Ebola virus (EBOV), Sudan virus (SUDV), Bundibugyo virus (BDBV), Taï Forest virus (TAFV), Reston virus (RESTV), and Marburg virus (MARV) to inhibit SeV-induced IFN-β promoter activity (Fig 1A). A titration (4 ng, 20 ng, and 100 ng) of each filovirus VP35-coding plasmid was transfected (Day 1), cells were mock-infected or infected with equal volumes of an SeV stock (Day 2) and luciferase expression was measured (Day 3). Each filoviral VP35 protein antagonized the SeV-induced expression of the IFN-β reporter in a dose-dependent manner (Fig 1A). This is in contrast to the samples in which no filoviral protein was expressed (white bar) or when MARV NP was expressed (green bars). Of the VP35 proteins from the five ebolavirus species, EBOV, SUDV, BDBV, RESTV and TAFV, all showed similar levels of IFN antagonism. The VP35 from RESTV exhibited a modest decrease in IFN-β reporter inhibition but was a stronger IFN antagonist than the VP35 from MARV (Fig 1A, gray and dark blue bars). This is consistent with previous reports that directly compared VP35 efficiencies of RESTV and MARV VP35 to EBOV VP35 [20,25]. Western blot analysis of cell lysates transfected with 100 ng of each expression construct indicates roughly equivalent expression of each VP35 (Fig 1B).

### The type I IFN antagonist activity of VP35 correlates with its ability to bind immunostimulatory SeV RNA

**Fig 2:**
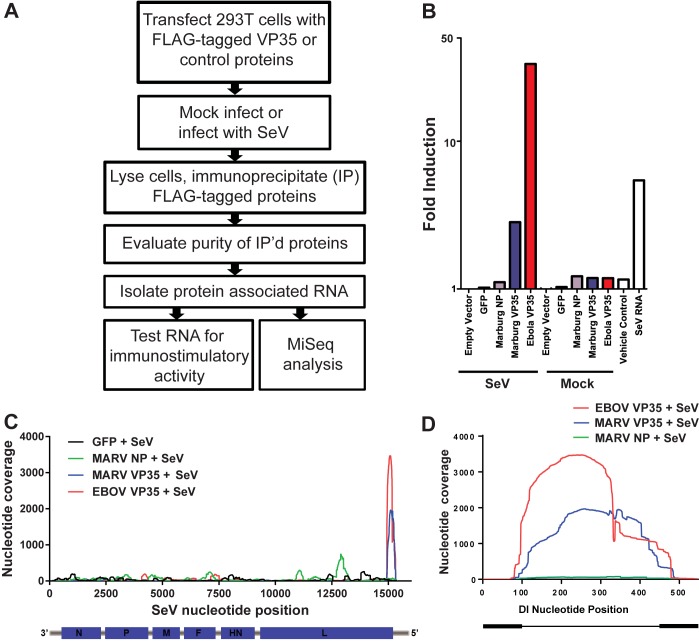
The type I IFN antagonist activity of VP35 correlates with its ability to bind the immunostimulatory Sendai virus defective interfering (DI) genome. (A) Overview of the methods used to characterize the RNA associated with VP35 in cells infected with SeV. (B) Immunostimulatory activity of RNA associated with FLAG-tagged filoviral proteins in the presence (left, “SeV Infected”) or absence (right, “Mock Infected”) of SeV infection. Cells were transfected with a plasmid expressing a luciferase reporter under the control of the ISG54 promoter and then transfected with the isolated RNA the following day. “Vehicle control” represents cells transfected with transfection reagent alone. “SeV RNA” represents cells transfected with viral RNA purified from the SeV stock. (C) Next-generation sequencing and mapping to the SeV genome. RNA associated with GFP, MARV NP protein, MARV VP35 protein, and EBOV VP35 was purified and subjected to Illumina Sequencing. The graph depicts nucleotide coverage (Y-axis) at each position of the SeV genome (X-axis). (D) Next-generation sequencing and mapping to the SeV DI genome. The same data from (C) was mapped to each nucleotide position of the SeV DI sequence. Thick black regions on each end of the DI diagram represent self-complementary sequences that presumably base-pair.

We hypothesized that isRNA species produced by SeV are actively bound by VP35, thus preventing their recognition by innate immune signaling pathways. To test this theory, RNA was isolated from purified VP35 proteins. Briefly, 293T cells were transfected with plasmids expressing FLAG-tagged MARV or EBOV VP35, MARV NP or GFP. Each group was either mock-infected or infected with equal volumes of an SeV stock one day post-transfection and lysed two days post-transfection (Fig 2A). FLAG-tagged VP35 or control proteins were immunoprecipitated with an anti-FLAG antibody and eluted with 3XFLAG peptide. SDS-PAGE and protein staining confirmed the purity of the precipitated proteins (data not shown). Next, RNAs bound to each purified protein were evaluated for immunostimulatory activity (Fig 2B). 293T cells were transfected with a luciferase reporter construct under the control of the interferon stimulated gene 54 (ISG54) promoter. The following day, equal volumes of the eluted RNA recovered from each purified protein were transfected into cells and the reporter activity was measured 18 hours post-transfection. The RNAs isolated from beads alone, GFP, and MARV NP in the presence or absence of SeV infection did not activate the ISG54 promoter (Fig 2B, left and right side). Strikingly, ISG54 promoter activity was detected following transfection of RNA recovered from EBOV VP35 and to a lesser extent, MARV VP35, in the presence of SeV infection (Fig 2B, left side, blue and red bars). Conversely, RNAs isolated from all purified proteins in the absence of SeV infection failed to induce luciferase expression from the ISG54 promoter (Fig 2B, “Mock”). A vehicle control (transfection reagent only) and purified SeV RNA from the input stock were used as negative and positive controls for ISG54 promoter activity, respectively (Fig 2B, white bars, right side). These data are consistent with a model in which the isRNAs generated during SeV infection are associated with VP35 in the infected cells of the established SeV reporter assay.

### EBOV and MARV VP35 bind RNA species at the 5’ end of the SeV genome

To identify the specific isRNAs bound by VP35, RNAs isolated from the immunoprecipitated FLAG-tagged proteins (Fig 2B) were sequenced on an Illumina MiSeq following an established sequence-independent single-primer amplification (SISPA) library construction method [26–28]. When mapping the resulting sequencing reads from EBOV and MARV VP35 samples to the SeV genome, most mapped to the 5’ end of the SeV genome, corresponding to the sequence of the copy-back DI RNA (Fig 2C, red and blue lines). Strikingly, these results resemble data from a prior study that identified SeV RNAs bound to RIG-I following SeV infection [23]. RNAs isolated from additional control transfections with GFP, and Marburg NP displayed reduced SeV genome coverage that was randomly distributed across the genome (Fig 2C, black and green lines). To confirm that the sequencing reads represent the DI genome of SeV, we directly mapped the reads obtained from the RNAs associated with MARV NP, and EBOV and MARV VP35s to the previously reported 546 nt-long copy-back DI sequence of SeV (Fig 2D). These data indicate that the ability of VP35 to suppress the IFN-β response in our assay is attributed to its ability to bind and sequester the immunostimulatory DI RNA of SeV.

### Basic residues in the VP35 dsRNA-binding domain are critical for binding SeV isRNA

**Fig 3:**
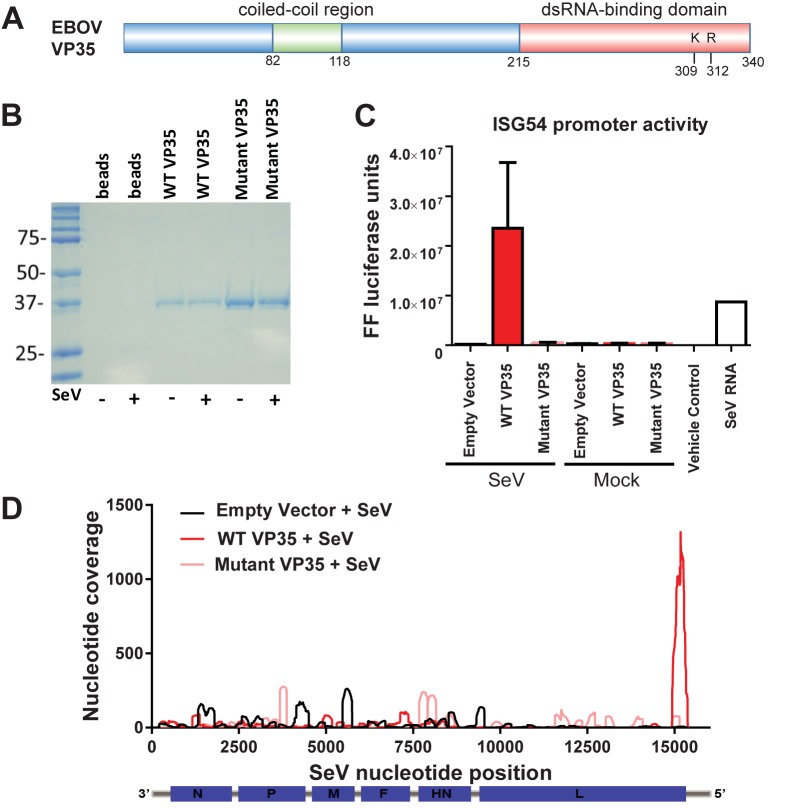
Mutations in VP35 important for dsRNA binding abrogate its ability to bind the immunostimulatory SeV DI. (A) Schematic of the EBOV VP35 protein containing a coiled-coil domain important for oligomerization in the N-terminal half of the protein and dsRNA-binding domain in the C-terminal half. Residues K309 and R312 were mutated to alanine to generate the EBOV VP35 RNA-binding mutant. (B) Protein staining of immunoprecipitated FLAG-tagged wild-type and mutant VP35 from which the RNA transfected in (C) and sequenced in (D) were recovered. (C) Immunostimulatory activity of RNA following immunoprecipitation of the pCAGGS empty vector (EV), wild-type EBOV VP35, and mutant EBOV VP35 in cells infected with SeV or mock-infected. (D) Next-generation sequencing and read mapping to the SeV genome. RNA associated with the pCAGGS empty vector, wild-type EBOV VP35, and mutant EBOV VP35 was purified and subjected to Illumina sequencing and the resulting reads were mapped to the SeV genome. The graph depicts nucleotide coverage (Y-axis) at each position of the SeV genome (X-axis).

To couple VP35 RNA binding to its ability to bind and sequester isRNA and prevent the activation of the IFN response, we directly compared the RNAs bound by both wild-type EBOV VP35 and a VP35 RNA-binding mutant in which two basic residues (K309 and R312) were substituted with alanine (Fig 3A). This (K309A/R312A) VP35 mutant has previously been shown to be attenuated for dsRNA-binding and IFN-α/β antagonist activity [16,24,29]. Empty pCAGGS vector, wild-type, and mutant FLAG-VP35s were transfected into 293T cells in triplicate. Twenty-four hours post-transfection, cells were either mock-infected or SeV-infected and each protein was purified. Gel analysis confirmed the size and purity of the immunoprecipitated wild-type and mutant VP35s (Fig 3B). RNAs isolated from the purified proteins were then transfected into the ISG54-promoter luciferase reporter assay. RNA isolated from cells transfected with wild-type VP35 and infected with SeV stimulated the ISG54 promoter (Fig 3C, red bar, left side). However, RNAs isolated from cells transfected with the mutant VP35 and infected with SeV were unable to induce ISG54 promoter activity (Fig 3C, pink bar, left side). As expected, RNA associated with both wild-type and mutant VP35 in uninfected cells failed to induce the ISG54 promoter (Fig 3C, right side “Mock”). These data clearly indicate the requirement for basic residues in the C-terminal dsRNA-binding domain of VP35 to bind SeV DI isRNAs.

We next deep sequenced the isolated RNA from the wild-type and mutant VP35 proteins and mapped sequencing reads to the SeV genome. Triplicate samples from each condition were combined and the total coverage across the SeV genome is presented. Sequencing reads from RNA obtained from samples transfected with wild-type VP35 were enriched at the 5’ end of the SeV genome, correlating with the SeV DI RNA (Fig 3D, red line). Moreover, coverage plots indicated that the SeV DI RNA binding in the VP35 (K309A/R312A) mutant was dramatically reduced compared to that of the wild-type VP35 (Fig 3D, pink line) and was similar to the background levels observed for the reads obtained from the RNA isolated from the empty pCAGGS vector-transfected cells (Fig 3D, black line). These data indicate that basic residues in VP35 required for dsRNA binding *in vitro* are critical for binding the immunostimulatory SeV DI RNA in infected cells.

### RNA-seq analysis identifies select host mRNAs preferentially bound by EBOV VP35

**Fig 4:**
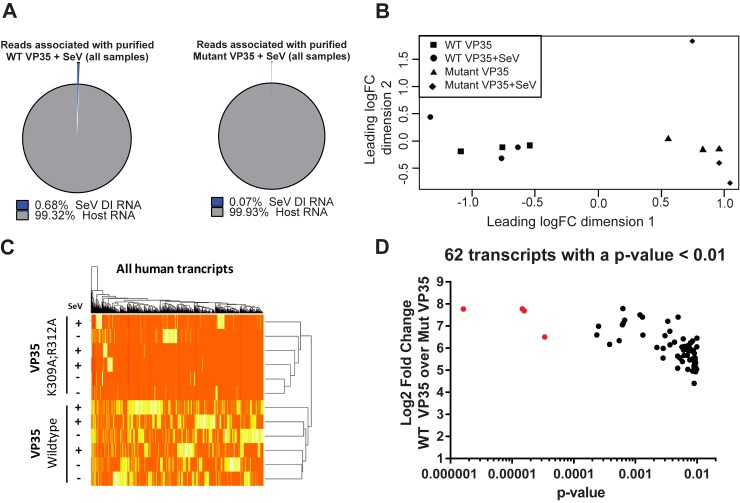
An analysis of host RNAs highlights transcripts bound by VP35. (A) Total number of sequencing reads from triplicate samples of wildtype and mutant VP35 proteins infected in the SeV infected groups. The pie charts depict the percentage of reads that map to the SeV DI genome and the percentage of reads that do not map to the SeV DI. (B) Multidimensional scaling (MDS) plot of triplicate samples from wild-type VP35 (black squares), wild-type VP35 infected with SeV (black circles), mutant VP35 (black triangle) and mutant VP35 infected with SeV (black diamond). Axes in the MDS plot (Leading logFC dim1 and Leading logFC dim2) are arbitrary, and the values on the axes are distance units. (C) Heat map showing the binding of the wild-type and mutant VP35 protein to human mRNA transcripts for all samples included in the analysis. Each row represents an experimental replicate, and each column represents a single transcript. Colors indicate relative abundance for each gene, where orange is low abundance and white is high abundance. (D) Sorting of the 62 most statistically significantly enriched mRNAs associated with VP35 (from the mock-infected wild-type VP35 samples). Y-axis denotes the p-value of each sample and X-axis denotes fold-change of transcript abundance between wild-type and mutant VP35.

Previous work has suggested that VP35 is a dsRNA-binding protein but does not bind cellular RNAs [30]. However, due to the nature of RNA to form secondary structures in cells [31], we evaluated the RNAs bound by both wild-type and mutant VP35 that did not map to the SeV genome. Performing the transfections, infections and pull-downs in triplicate allowed us to perform statistical analyses on the binding differences between the wild-type VP35 and the corresponding RNA-binding mutant. In addition, we analyzed host RNAs bound both in the absence and presence of SeV to determine if SeV infection influenced VP35 host RNA binding. While reads from the wild-type VP35 samples were approximately 10-fold enriched in SeV RNA compared to the mutant VP35 sample, over 99% of the reads in both samples did not map to the SeV genome (Fig 4A, reads from triplicate samples were combined). RNA sequences from wild-type and mutant VP35 pull-down experiments were then mapped to the *Homo sapiens GRCh38* transcriptome to determine host RNAs bound by EBOV VP35. Strikingly, wild-type VP35 was found to have a unique host RNA-binding signature compared to the mutant VP35, in either the presence or absence of SeV infection, as analyzed by multidimensional scaling (MDS), an analysis that visualizes the level of similarity between all experimental data points (Fig 4B). A global heat map illustrating binding patterns across the entire transcriptome between wild-type and mutant VP35 confirmed that all wild-type samples cluster independently from mutant VP35 in both the presence and absence of SeV infection (Fig 4C). During this analysis, 62 RNAs were found to be significantly enriched (p<0.01) in the wild-type VP35 sample over the mutant VP35 sample (Fig 4D, black dots and S1 Table), but only four RNAs were found to be significantly enriched with a low false discovery rate (p<0.01, FDR<0.1). These four cellular RNAs (Fig 4D, red dots) include the transcripts that code for PAICS (ENSG00000128050.6), EEF1A1 (ENSG00000156508.15), UBAP2L (ENSG00000143569.16), and MDM2 (ENSG00000135679.19). Secondary structure prediction of the most enriched host transcript, PAICS, yields a molecule with a long stable stem similar to the stem of the SeV DI (S1 Fig). Future work is required to determine if VP35 preferentially binds these host RNAs because of RNA secondary structure similar to the panhandle structure of the SeV DI.

### Multiple filovirus VP35 proteins, inducing a 2014 outbreak sequence binds the immunostimulatory SeV DI RNA

**Fig 5:**
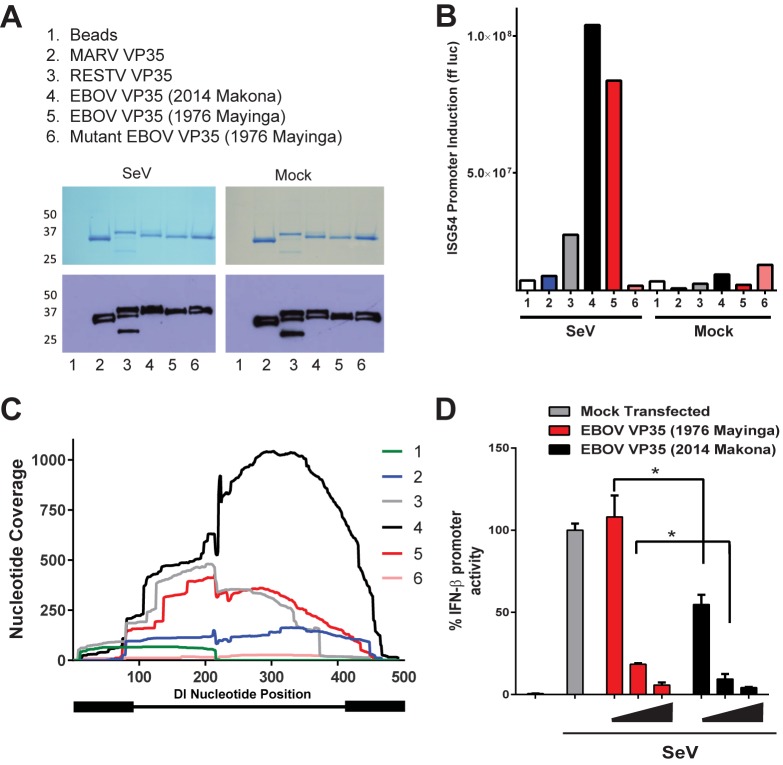
The 2014 EBOV strain and other filovirus VP35 proteins bind the immunostimulatory SeV DI. (A) A key describing each of the experimental conditions tested with corresponding numbers for panels A-C. “Beads” refers to the control sample in which cells that were transfected with empty pCAGGS vector only. Protein stain (top) and immunoblot against FLAG tag (bottom) of the immunoprecipitated supernatant showing the purity and relative expression levels of the VP35 proteins from MARV, RESTV, EBOV 1976 (Mayinga), EBOV 2014 (Makona) and the VP35 EBOV RNA-binding mutant (Mayinga). (B) Ability of RNA purified from each VP35 in the presence (left) or absence (right) of SeV infection to stimulate the ISG54 promoter. (C) Next-generation sequencing and mapping to the SeV DI genome. RNA associated with each VP35 variant was purified and subjected to Illumina sequencing. The graph depicts nucleotide coverage (Y-axis) at each position of the SeV DI genome (X-axis). Thick black regions on each end of the DI diagram represent self-complementary sequences that presumably base-pair. (D) Comparison of IFN-β inhibition by the Mayinga and Makona EBOV VP35 proteins from the 1976 and 2014 outbreaks, respectively. A * between groups denotes the results of a T-test where p <0.05.

The recent EBOV outbreak in West Africa was the largest in history, and we therefore sought to compare the ability of the 2014 Makona EBOV VP35 to bind isRNA relative to other filovirus VP35 proteins, despite only differing from Mayinga EBOV (1976) VP35 by 4 amino acids (A12V, S41N, T68M and N204D) [2]. We directly compared the VP35 from the Makona strain to the VP35s from 1976 Mayinga EBOV, RESTV, and MARV. We chose RESTV and MARV VP35 since they are demonstrated be less efficient inhibitors of SeV IFN induction compared to EBOV VP35 [20,25]. RNA was isolated from equivalent amounts of each purified VP35 protein (Fig 5A). Analysis of RNA bound to Makona EBOV VP35 in SeV-infected cells confirmed its ability to bind isRNA at least as efficiently as Mayinga EBOV VP35, and more efficiently than both RESTV and MARV VP35 (Fig 5B). The immunostimulatory activity of RNA bound by each VP35 was also reflected in our deep sequencing analysis. This demonstrated that the VP35 from the Makona strain most efficiently bound the SeV DI RNA (Fig 5C). MARV also bound the SeV DI, but at levels lower than the VP35 from EBOV and RESTV species (Fig 5C, compare blue to red and gray lines). Finally, a direct comparison between the Mayinga and Makona VP35s indicated both EBOV VP35s inhibit the SeV IFN-β reporter assay, but the Makona strain inhibited IFN-β transcription more efficiently at equivalent doses (Fig 5D, red and black bars). Our data suggests that the Makona strain of VP35 is a potent inhibitor of the host IFN signaling pathway, consistent with previous reports [32]. While our SeV DI RNA-binding and reporter assays are only semi-quantitative, they do suggest that the 2014 Makona VP35 is at least as efficient, if not more efficient, at binding isRNA and inhibiting IFN-β reporter activity compared to the 1976 Mayinga VP35. A model summarizing these data suggests VP35 sequesters isRNA in virus-infected cells to inhibit RIG-I like receptor (RLR) activation and also binds hosts RNAs (S2 Fig).

## Discussion

Work from our study provides novel insight into how filovirus VP35 proteins bind and sequester isRNA in infected cells. Cellular pattern recognition receptors (PRR) detect pathogen-associated molecular patterns (PAMPs) such as viral proteins and viral RNA [33]. Recognition of viral RNA, by the cytoplasmic family of PRRs called RIG-I-like receptors (RLRs) which include RIG-I and MDA5, leads to transcription of IFN-β and subsequent activation of IFN stimulated genes, or ISGs. Conversely, viruses have evolved strategies to evade and antagonize RLR antiviral immune responses, and VP35 is a well-documented antagonist of type I IFN induction [5,6,13,15–17,19,20,24,34]. VP35 can inhibit RIG-I activation by directly interacting with the kinases TBK-1 and IKKε [35]. Moreover, the VP35 IFN antagonist activity strongly correlates with its ability to bind dsRNA, since mutations in VP35 that abolish RNA binding attenuate its ability to inhibit IFN production and render recombinant viruses avirulent in animal models [16,19]. While VP35 RNA-binding activity has previously been shown to be important for IFN antagonist activity, there are no reports describing the RNA ligands bound by VP35 in virus-infected cells.

In this report, we utilized an established SeV IFN-β reporter assay to demonstrate that Ebola and Marburg VP35 proteins specifically bind viral isRNAs defective interfering (DI) SeV genomes, in cell culture. To our knowledge this is the first study to directly compare the ability of VP35 from all 5 species of *Ebolavirus* and a *Marburgvirus* to inhibit IFN-β production. Furthermore, we report a correlation between the ability of specific filovirus VP35 proteins to bind the DI SeV genome and their ability to inhibit SeV-mediated IFN-β promoter activity. Our deep sequencing analysis of the RNAs associated with Ebola VP35 also uncovered select host RNAs that are significantly enriched in WT samples over the RNA-binding mutant samples. Taken together, these are the first data to demonstrate that VP35 binds isRNA during viral infection and is the first study to show preferential binding of certain host RNA by VP35.

Our work builds on several studies that clearly demonstrate the correlation between VP35 dsRNA-binding activity and its ability to inhibit type I IFN responses [16,20,25]. RIG-I, the cellular dsRNA-sensing protein, has been shown to prefer dsRNA substrates containing 5’ -triphosphate, and to a lesser extent 5’-diphosphate ends [36,37]. Using *in vitro* synthesized dsRNAs, it was previously demonstrated that the optimal RIG-I ligand is a 5’ ppp dsRNA of at least 10 bp in length [38,39]. Next-generation sequencing revealed that during infection with SeV, RIG-I preferentially binds the defective interfering genomes of the virus [23]. Furthermore, Baum *et al*. showed that during SeV infection, RIG-I specifically binds short subgenomic SeV (DI) RNAs [23]. Although Ebola virus VP35 is known to inhibit RIG-I-mediated type I IFN response through a dsRNA-binding-dependent mechanism, a dsRNA substrate bound by VP35 during a viral infection had not been demonstrated before and in this study we provide evidence that VP35 binds RNA species recognized by RIG-I which may serve to mask immune activation following viral infection.

Our work utilizes SeV (Cantell strain), which serves as a tool to study filoviral VP35-mediated IFN antagonism, and has been demonstrated to generate elevated levels of defective interfering (DI) genomes [21,22]. Although the generation of DIs was once believed to be an artifact of continuous passage of virus in cell culture, it is now becoming apparent that DIs may be an inevitable byproduct of virus replication and have been reported in almost all families of RNA viruses [40,41]. Although the presence of DIs in natural filovirus infection has yet to be reported, EBOV-infected Vero cells produce DIs that can be detected as early as the second passage [42]. Recently, it was shown that respiratory syncytial virus (RSV) infection generates the production of DIs in human patients at levels that were directly correlated with antiviral gene expression, illustrating their significance [43]. Therefore, it is possible VP35 functions, in part, to bind filovirus copy-back DI genomes generated during infection to prevent innate immune activation during infection.

One of the most striking results from our work is the ability of EBOV VP35 to preferentially bind select host RNAs. While we identify ~60 host RNAs significantly enriched for binding by WT VP35 over a VP35 RNA-binding mutant; 4 of these had a false discovery rate less than 0.1, implicating their significance (Fig 4 and S1 Table). Strikingly, the most enriched host RNA (PAICS) bound to wild-type VP35 versus the mutant VP35 has a predicted stem-loop similar to the Sendai virus DI genome (S1 Fig). This could indicate that stem-loops play a role in VP35 RNA binding. RNA isolated from wild-type VP35 in the absence of SeV infection was not immunostimuatory in our assays. Since most host RNAs are capped, it is possible that these RNAs were bound due to secondary stem-loop structures that resemble that of the SeV DI but are not immunosimulatory since previous work has shown that the uncapped 5’ triphosphate of the SeV DI is critical for robust IFN activation [23]. Others have reported that RIG-I interacts with select host RNA species [44], suggesting that like VP35, RLRs bind host RNA with some specificity. However additional work indicates that ATP-hydrolysis activity of RIG-I functions to prevent recognition of self-RNA to prevent unintentional RIG-I signaling through prolonged RNA binding [45]. The significance of host RNA bound by VP35 remains a focus of future work.

Reported fatality rates from human filovirus infection vary widely. *Zaire ebolavirus* (EBOV) can result in fatality rates up to 90%, while infection with *Reston ebolavirus* is asymptomatic. The fatality rate for all known human Marburg infections is approximately 80% [46]. While some of these differences are almost certainly due to small sample sizes, imperfect diagnosis and reporting, and health service disparities, there is evidence that differences in virulence exist between filovirus species [20,25]. Since virulence is correlated to the ability of the virus to suppress the innate immune response and VP35 is one of the major viral players in suppressing the host innate immune response, our comparison of VP35 proteins from Marburgvirus and all five species of ebolavirus is informative. Moreover, we demonstrate the 2014 Makona VP35 works as efficiently as the 1976 Mayinga VP35 to bind immunostimulatory RNA and to antagonize IFN-β production in the SeV reporter assay, consistent with previous reports [32]. While our SeV DI RNA-binding and reporter assays are only semi-quantitatve, they do suggest that the 2014 Makona VP35 is at least as efficient, if not more efficient, at binding isRNA and inhibiting IFN-β reporter activity. MARV VP35 proved to be the weakest IFN antagonist of the filovirus VP35 in our assay, consistent with previous reports the EBOV VP35 more efficiently inhibits IFN responses [25]. This data is in line with prior work [20], and demonstrates the utility of our assay to rapidly evaluate filovirus IFN antagonist efficiency.

Taken together, our data support an established model where VP35 binds isRNA in cells to evade RIG-I like receptor (RLR) activation while also binding select host RNA (S2 Fig). There is significant interest to target VP35 activity with small molecule as a platform for antiviral approach [47–51], and our work helps further define the biological activity of this protein.

## Materials and methods

### Constructs

FLAG-tagged viral proteins were all N-terminally tagged in the pCAGGS vector backbone under control of the CMV promoter. SUDV (KU182912), BDBV (KU182911), RESTV (JX477166), and TAFV (KU182910) VP35 sequences were synthesized (Genewiz), amplified by PCR, and inserted into the pCAGGS-FLAG backbone using unique NotI and NheI restriction sites. Introduction of the K309A and R312A into EBOV (Mayinga) (KR0636671) VP35 was accomplished by overlapping PCR.

### Tissue culture, transfections, and infections

293T cells were maintained in DMEM (Gibco) supplemented with 10% fetal bovine serum (Hyclone) and stored in humidified incubators at 37°C and 5% CO_2_. DNA and RNA transfections were performed using TransIT-LT1 transfection reagent and TransIT-mRNA transfection kit (Mirus), respectively, according to manufacturer’s recommendations. For infections, the Cantell strain of Sendai virus was generated in 8-day-old embryonated chicken eggs and diluted 1:10 in infection media (DMEM, 0.2% BSA), added to cells for one hour, and then replaced with complete media (DMEM, 10% FBS). For a given experiment, equal volumes of the same SeV stock were used to infect each sample.

### ISG54 and IFN-β reporter assay

The Dual Luciferase Reporter Assay System (Promega) was used according to manufacturer’s instructions. Briefly,cells were lysed in passive lysis buffer and luciferase expression under the control of the IFN-β promoter was measured with a Glomax Multi Detection System (Promega). In IFN antagonist assay in which filoviral proteins expression was titrated, empty pCAGGS plasmid was used to keep total plasmid amount transfected constant and a plasmid expressing *Renilla* luciferase was used as a transfection control.

### Immunoprecipitations and RNA isolation

Cells transfected with FLAG-tagged filoviral proteins and vector controls were washed twice with PBS and lysed in NP-40 buffer containing protease inhibitors. Cell membranes were pelleted with high-speed centrifugation (10,000xG) for 5–10 minutes and the resulting supernatants were subjected to overnight incubation with anti-FLAG magnetic beads (Sigma Aldrich) at 4°C. The following day, the anti-FLAG beads were washed four times with cold NP-40 buffer containing protease inhibitors and FLAG-tagged proteins were eluted by displacement upon addition of an excess of FLAG peptide (Sigma Aldrich). Buffer RLT (Qiagen) was added to the supernatant containing the eluted FLAG-tagged proteins and RNA was isolated using the RNeasy kit (Qiagen). Immunoprecipitated proteins were resolved by electrophoresis on 10% Tris-Glycine polyacrylamide gels (Lonza). Protein gels were either stained with SimplyBlue SafeStain (Invitrogen) protein stain according to manufacturer’s recommendations or proteins were transferred to nitrocellulose (iBlot Gel Transfer System, Novex by Life Technologies) for western blotting and probed with mouse monoclonal anti-FLAG (M2) antibody (Sigma Aldrich) followed by HRP-conjugated goat anti-mouse IgG (MP).

### Library construction and sequencing

Sequence-independent single primer amplification (SISPA) was used to generate a representative sequencing library of RNAs bound to VP35 [26,27]. Briefly, RNAs isolated from anti-FLAG immunoprecipitated proteins were subjected to sequence-independent reverse transcription using bar-coded random hexamer primers. This cDNA was amplified further using PCR primers identical to the bar-code portion of the random hexamer primers used in initial reverse transcription reactions. Since each bar code was unique to each sample, the PCR products were purified using QIAquick PCR purification kit (Qiagen), pooled, and sequenced by MiSeq (Illumina).

### Bioinformatic analyses

MiSeq reads were trimmed with Trimmomatic v0.33 [52] and mapped to the *Homo sapiens* GRCh38 genome available from NCBI using Tophat [53,54]. Read counts per gene were calculated for each sample using HTSeq-count [55] and statistical evaluation was performed using the edgeR [56] package in R [57].

## Supporting information

S1 Table Host RNAs bound by wild-type EBOV VP35. Transcripts listed were significantly enriched in the wild-type VP35 samples compared to the RNA-binding mutant samples. Yellow cells denote host genes with a p-value of less than 0.01 and a False Discovery Rate of less than 0.1.
(PDF)

S1 Fig Secondary structure of the Sendai virus (SeV) and PAICS, the most significantly enriched host RNA bound by VP35, contain stable stem structures. RNA secondary structures of RNA molecules bound by EBOV VP35 protein were predicted using CLC Genomics Workbench 8.5 (QIAGEN Bioinformatics). (A) RNA secondary structure of the 546 nucleotide defective interfering (DI) genome of SeV. (B) RNA secondary structure of the 3,350 nucleotide host cell transcript PAICS (transcript variant 1, NM_001079525). (C) RNA secondary structure of the termini of the EBOV genome. The 5’ most 50 nucleotides and the 3‘ most 54 nucleotides were joined by adding ten “A”s (capital “A”s in left bulge). VP35 is required for EBOV genome packaging and is assumed to bind the genome (29). Panhandle structure of the EBOV termini has been previously predicted (J. Virol. 2005 Aug; 79(16): 10660–10671: PMID: 16051858). While we do not directly show VP35 binding this EBOV genomic region in our study, it represents a viral stem loop predicted to be present during EBOV infection which could serve as a binding target of VP35.
(EPS)

S2 Fig Model of VP35-mediated evasion of innate immune responses. Viral infection leads to the generation of dsRNA that can be recognized by RLR (RIG-I-like receptors) leading to a signal cascade that results in the induction of IFNs. Immunostimulatory viral dsRNAs are also recognized and bound by VP35 sequestering them away from RLRs and preventing the activation of the innate immune response. Our data also observed that EBOV VP35 specifically binds a subset of cellular RNAs in host cells.
(PDF)
