# Supplementary material for: The Ebola virus VP35 protein binds viral immunostimulatory and host RNAs identified through deep sequencing
Source: PLoS One. 2017 Jun 21;12(6):e0178717. doi: 10.1371/journal.pone.0178717 (PMC5479518; doi:10.1371/journal.pone.0178717)
Supplement: S1 Table — Transcripts listed were significantly enriched in the wild-type VP35 samples compared to the RNA-binding mutant samples. Yellow cells denote host genes with a p-value of less than 0.01 and a False Discovery Rate of less than 0.1. (PDF) [file pone.0178717.s001.pdf]

**S1 Table. Host RNAs bound by wild-type EBOV VP35. Transcripts listed were significantly enriched in the wild-type VP35 samples compared to the RNA-binding mutant samples**
**Yellow cells denote host genes with a p-value of less than 0.01 and a False Discovery Rate of less than 0.1.**

| Gene name | Gene ID | log2 Fold Change (WT VP35/ Mutant VP35 | p-value (cut off at 0.01) | False Discovery Rate (FDR less than 0.1 highlighted yellow) | Gene description |
|---|---|---|---|---|---|
| PAICS | ENSG00000128050.6 | 7.772489755 | 1.62E-06 | 0.004680565 | Phosphoribosylaminoimidazole Carboxylase, Phosphoribosylaminoimidazole Succinocarboxamide Synthetase |
| EEF1A1 | ENSG00000156508.15 | 7.774185844 | 1.46E-05 | 0.015133307 | Elongation factor 1-alpha 1 (EF-1-alpha-1)(Elongation factor 1 A-1)(eEF1A-1)(Elongation factor Tu)(EF-Tu)(Leukocyte receptor cluster member 7) |
| UBAP2L | ENSG00000143569.16 | 7.692490751 | 1.57E-05 | 0.015133307 | Ubiquitin associated protein 2-like (HGNC Symbol) |
| MDM2 | ENSG00000135679.19 | 6.503100354 | 3.38E-05 | 0.024433529 | E3 ubiquitin-protein ligase Mdm2 (EC 6.3.2.-)(p53-binding protein Mdm2)(Oncoprotein Mdm2)(Double minute 2 protein)(Hdm2) |
| MAGT1 | ENSG00000102158.17 | 6.598189639 | 0.000236413 | 0.121959595 | Magnesium transporter 1 |
| NUCKS1 | ENSG00000069275.12 | 6.986755582 | 0.000253291 | 0.121959595 | Nuclear casein kinase and cyclin-dependent kinase substrate 1 |
| MIER1 | ENSG00000198160.12 | 6.171916443 | 0.000381046 | 0.157263095 | Mesoderm induction early response 1 homolog |
| RP1-177G6.1 | ENSG00000203930.8 | 6.334063976 | 0.000546166 | 0.16159546 | Long intergenic non-protein coding RNA 632 |
| DDTL | ENSG00000099974.7 | 7.059726346 | 0.000615497 | 0.16159546 | D-Dopachrome Tautomerase-Like Protein |
| MGRN1 | ENSG00000102858.10 | 7.787300099 | 0.000626225 | 0.16159546 | Mahogunin, ring finger 1 |
| TMPO | ENSG00000120802.11 | 7.117096885 | 0.000633263 | 0.16159546 | Thymopoietin |
| STAP2 | ENSG00000178078.9 | 7.261024837 | 0.000671217 | 0.16159546 | Signal transducing adaptor family member 2 isoform 1 |
| STAG3L3 | ENSG00000174353.15 | 7.502386323 | 0.001184484 | 0.254923943 | Stromal antigen 3-like 3 |
| FOXRED2 | ENSG00000100350.12 | 7.40080848 | 0.00130187 | 0.254923943 | FAD-dependent oxidoreductase domain-containing protein 2 Precursor |
| XIST | ENSG00000229807.7 | 6.591526539 | 0.001323593 | 0.254923943 | X (inactive)-specific transcript (non-protein coding) |
| RILPL1 | ENSG00000188026.9 | 6.031174686 | 0.002235718 | 0.297376737 | RILP-like protein 1 (Rab-interacting lysosomal-like protein 1) |
| ACTG1 | ENSG00000184009.7 | 5.547291314 | 0.002809362 | 0.297376737 | Actin gamma 1 |
| CTDSPL2 | ENSG00000137770.11 | 5.991502718 | 0.002832788 | 0.297376737 | CTD (carboxy-terminal domain, RNA polymerase II, polypeptide A) small phosphatase like 2 |
| EIF4G3 | ENSG00000075151.17 | 6.566576616 | 0.003017884 | 0.297376737 | Eukaryotic translation initiation factor 4 gamma 3 |
| SRRM1 | ENSG00000133226.14 | 7.216633989 | 0.003356452 | 0.297376737 | Serine/arginine repetitive matrix protein 1 |
| SRP9 | ENSG00000143742.10 | 6.764763938 | 0.003623792 | 0.297376737 | Signal recognition particle 9kDa |
| NPM1 | ENSG00000181163.11 | 6.148051428 | 0.003653284 | 0.297376737 | Nucleophosmin |
| ZSWIM1 | ENSG00000168612.4 | 6.27053932 | 0.004325236 | 0.297376737 | Zinc finger, SWIM-type containing 1 |
| RPL37 | ENSG00000145592.11 | 5.634422799 | 0.004847204 | 0.297376737 | Ribosomal protein L37 |
| GPT2 | ENSG00000166123.11 | 5.088543364 | 0.00485981 | 0.297376737 | Glutamate pyruvate transaminase 2 |
| LIMD1 | ENSG00000144791.7 | 7.401369734 | 0.004928175 | 0.297376737 | LIM domains containing 1 |
| TBL1XR1 | ENSG00000177565.13 | 5.55087443 | 0.005279995 | 0.297376737 | F-box-like/WD repeat-containing protein TBL1XR1 |
| SRSF4 | ENSG00000116350.13 | 6.048874303 | 0.005700966 | 0.297376737 | Serine/Arginine-Rich Splicing Factor 4 |
| NOL9 | ENSG00000162408.10 | 5.388411502 | 0.00579013 | 0.297376737 | Nucleolar protein 9 |
| DCAF16 | ENSG00000163257.8 | 6.418020566 | 0.005963036 | 0.297376737 | DDB1 And CUL4 Associated Factor 16 |
| NUP155 | ENSG00000113569.13 | 5.919344752 | 0.006038805 | 0.297376737 | Nucleoporin 155kDa |
| LRPPRC | ENSG00000138095.16 | 5.673206266 | 0.00612877 | 0.297376737 | Leucine-rich PPR motif-containing protein, mitochondrial Precursor |
| RPS16 | ENSG00000105193.6 | 6.079768352 | 0.006256834 | 0.297376737 | 40S ribosomal protein S16 |
| HSD17B14 | ENSG00000087076.6 | 6.072810975 | 0.006764263 | 0.297376737 | 17-beta-hydroxysteroid dehydrogenase 14 |
| RBM6 | ENSG00000004534.12 | 5.672092639 | 0.006939973 | 0.297376737 | RNA binding motif protein 6 |
| ZMYM1 | ENSG00000197056.7 | 5.03713702 | 0.007072434 | 0.297376737 | Zinc finger, MYM-type 1 |
| PDS5A | ENSG00000121892.12 | 6.11285061 | 0.007126884 | 0.297376737 | Sister chromatid cohesion protein PDS5 homolog A |
| TBC1D14 | ENSG00000132405.16 | 5.860596907 | 0.007273659 | 0.297376737 | TBC1 domain family member 14 |
| SENP1 | ENSG00000079387.11 | 5.876774665 | 0.007529218 | 0.297376737 | Sentrin-specific protease 1 |
| YTHDF3 | ENSG00000185728.14 | 6.019639443 | 0.007590554 | 0.297376737 | YTH domain family protein 3 |
| SSR3 | ENSG00000114850.4 | 5.462562081 | 0.007666455 | 0.297376737 | Signal sequence receptor, gamma (translocon-associated protein gamma) |
| MLXIP | ENSG00000175727.11 | 6.236163239 | 0.007822281 | 0.297376737 | MLX interacting protein |
| RPS6KB1 | ENSG00000108443.11 | 6.253494347 | 0.008061427 | 0.297376737 | Ribosomal protein S6 kinase beta-1 |
| CXorf56 | ENSG00000018610.11 | 5.881429131 | 0.008200732 | 0.297376737 | Chromosome X open reading frame 56 |
| SOAT1 | ENSG00000057252.10 | 5.509490572 | 0.008398197 | 0.297376737 | Sterol O-acyltransferase 1 |
| SLC16A1 | ENSG00000226419.4 | 6.322519739 | 0.008473716 | 0.297376737 | Solute carrier family 16(Monocraboxylate Transporter), member 1 |
| AMD1 | ENSG00000123505.12 | 4.965100678 | 0.008518415 | 0.297376737 | S-adenosylmethionine decarboxylase proenzyme |
| LSM14A | ENSG00000257103.6 | 5.894020561 | 0.008737236 | 0.297376737 | LSM14A MRNA Processing Body Assembly Factor |
| ACACA | ENSG00000278540.2 | 5.168445787 | 0.008938589 | 0.297376737 | Acetyl-CoA carboxylase alpha |
| DMTF1 | ENSG00000135164.16 | 5.199575805 | 0.008939507 | 0.297376737 | Cyclin D binding myb-like transcription factor 1 |
| ABL2 | ENSG00000143322.17 | 4.403346491 | 0.008991151 | 0.297376737 | Tyrosine-protein kinase ABL2 |
| ATF7IP | ENSG00000171681.10 | 5.38644173 | 0.009011743 | 0.297376737 | Activating transcription factor 7 interacting protein |
| KLHL26 | ENSG00000167487.9 | 5.760448756 | 0.009044621 | 0.297376737 | Kelch-Like Family Member 26 |
| ZNF292 | ENSG00000188994.10 | 5.430540118 | 0.009285781 | 0.297376737 | Zinc finger protein 292 |
| COX7A2L | ENSG00000115944.12 | 5.538155044 | 0.009310472 | 0.297376737 | Cytochrome c oxidase subunit VIIa polypeptide 2-like protein |
| SMC3 | ENSG00000108055.9 | 5.549982782 | 0.009344435 | 0.297376737 | Structural maintenance of chromosomes 3 |
| SEP15 | ENSG00000183291.13 | 4.927670806 | 0.009474717 | 0.297376737 | 15 KDa Selenoprotein |
| CCT8 | ENSG00000156261.10 | 5.323938536 | 0.009505394 | 0.297376737 | T-complex protein 1 subunit theta |
| NIPBL | ENSG00000164190.14 | 5.114880435 | 0.009533064 | 0.297376737 | Nipped-B-like protein |
| EIF4G2 | ENSG00000110321.13 | 5.044367551 | 0.009836729 | 0.297376737 | Eukaryotic translation initiation factor 4 gamma, 2 |
| STT3A | ENSG00000134910.10 | 6.056054055 | 0.009912791 | 0.297376737 | Subunit of the oligosaccharyltransferase complex |
| ZNF714 | ENSG00000160352.13 | 6.447914495 | 0.00999696 | 0.297376737 | Zinc finger protein 714 |
