# Supplementary material for: The Ebola virus VP35 protein binds viral immunostimulatory and host RNAs identified through deep sequencing
Source: PLoS One. 2017 Jun 21;12(6):e0178717. doi: 10.1371/journal.pone.0178717 (PMC5479518; doi:10.1371/journal.pone.0178717)
Supplement: S2 Fig — Viral infection leads to the generation of dsRNA that can be recognized by RLR (RIG-I-like receptors) leading to a signal cascade that results in the induction of IFNs. Immunostimulatory viral dsRNAs are also recognized and bound by VP35 sequestering them away from RLRs and preventing the activation of the innate immune response. Our data also observed that EBOV VP35 specifically binds a subset of cellular RNAs in host cells. (PDF) [file pone.0178717.s003.pdf]

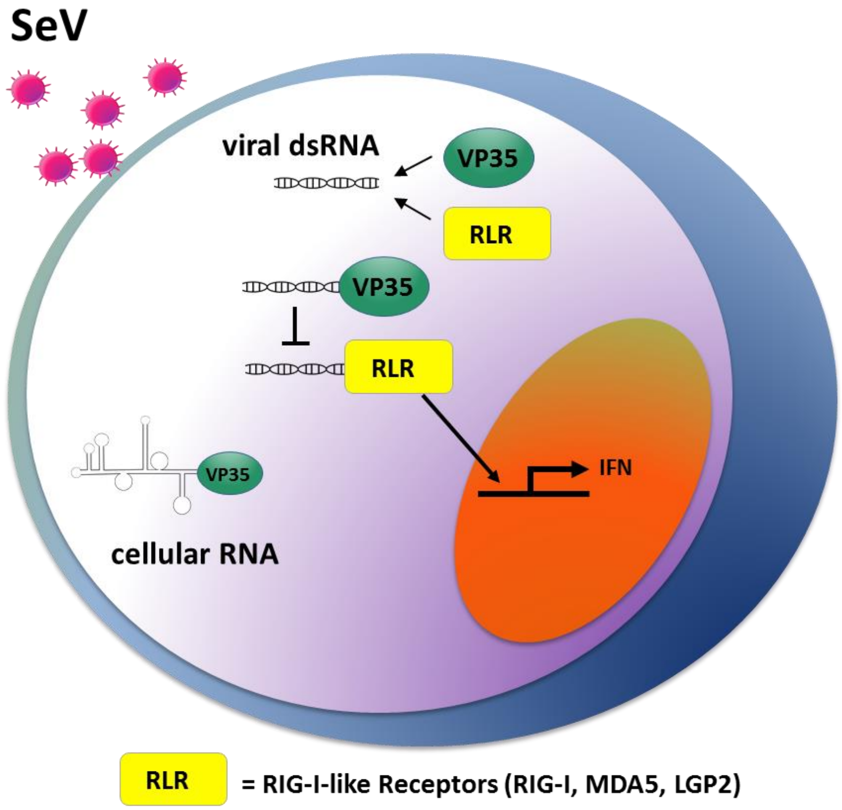

Fig. 6. Model of VP35-mediated evasion of innate immune responses. Viral infection leads to the generation of dsRNA that can be recognized by RLR (RIG-I-like receptors) leading to a signal cascade that results in the induction of IFNs. Immunostimulatory viral dsRNAs are also recognized and bound by VP35 sequestering them away from RLRs and preventing the activation of the innate immune response. We also observed that EBOV VP35 specifically binds a subset of cellular RNAs.
